# Electrophysiological, Morphologic, and Transcriptomic Profiling of the Ogura-CMS, DGMS and Maintainer Broccoli Lines

**DOI:** 10.3390/plants11040561

**Published:** 2022-02-21

**Authors:** Zhansheng Li, Lixiao Song, Yumei Liu, Fengqing Han, Wei Liu

**Affiliations:** 1 Key Laboratory of Biology and Genetic Improvement of Horticultural Crops, Institute of Vegetables and Flowers, Chinese Academy of Agricultural Sciences, Ministry of Agriculture, #12 Zhong Guan Cun Nandajie Street, Beijing 100081, China; liuyumei@caas.cn (Y.L.); hanfengqing@caas.cn (F.H.); 2 China Vegetable Biotechnology (Shouguang) Co., Ltd., Shouguang 262700, China; liuwei01@caas.cn; 3 Institute of Agricultural Resources and Environment, Jiangsu Academy of Agricultural Sciences, Nanjing 210014, China; prettygirl_slx@163.com

**Keywords:** broccoli, cytoplasmic male sterility, dominant genic male sterility, agronomy, RNA-sequencing

## Abstract

To better serve breeding of broccoli, the electrophysiological, morphological and transcriptomic profiling of the isogenic Ogura-CMS, DGMS and their maintainer fertile lines, were carried out by scanning electron microscopy, investigation of agronomic traits and RNA-sequencing analysis. The agronomic traits of plant height, length of the largest leaf, plant spread angle, single head weight, head width and stem diameter showed stronger performance in Ogura-CMS broccoli than in DGMS line or maintainer fertile line. However, the Ogura-CMS broccoli was poorer in the seed yield and seed germination than in the DGMS line and maintainer fertile line. Additionally, the DGMS broccoli had longer maturation and flowering periods than the Ogura-CMS and maintainer fertile lines. There were obvious differences in the honey gland, happening in the male sterility and fertile lines of broccoli. Additionally, the mechanism regulating Ogura-CMS and DGMS in broccoli was investigated using florets transcriptome analyses of the Ogura-CMS, DGMS and maintainer fertile lines. As a result, a total of 2670 differentially expressed genes (DEGs) were detected, including 1054 up- and 1616 downregulated genes in the Ogura-CMS and DGMS lines compared to the maintainer fertile line. A number of functionally known genes involved in plant hormones (auxin, salicylic acid and brassinosteroid), five Mitochondrial Oxidative Phosphorylation (OXPHOS) genes of *atp8*, *LOC106319879*, *LOC106324734*, *LOC106314622* and *LOC106298585*, and three upregulated genes (*Lhcb1*, *Lhcb3* and *Lhcb5*) associated with the photosynthesis-antenna protein pathway, were obviously detected to be highly associated with reproductive development including flowering time, maturity and reproductive period in the Ogura-CMS and DGMS broccoli comparing to their maintainer fertile line. Our research would provide a comprehensive foundation for understanding the differences of electrophysiological, morphological and transcriptomic profiles in the Ogura-CMS, DGMS and maintainer broccoli, and as well as being beneficial to exploring the mechanism of male sterility in *Brassica* crops.

## 1. Introduction

Broccoli (*Brassica oleracea* L. *var. italica*) is a well-known vegetable grown worldwide that belongs to the cruciferous vegetable family, which includes cabbage, kale, cauliflower, Brussels sprouts, Chinese cabbage, rutabaga and turnips. Broccoli contains many nutrients and few calories [1], exhibiting anticancer [2], weight loss promoting [3], cardiovascular and cerebrovascular disease-preventing properties that are beneficial to human health [4,5]. In 2017, the area in China under cultivation for broccoli has exceeded 70,000 ha based on data from the Ministry of Agriculture [6,7].

Currently, commercial broccoli crops primarily consist of hybrid cultivars benefiting from heterosis, and the hybrid cultivars are mostly male sterility lines, especially Ogura cytoplasmic male sterility (Ogura-CMS) lines. The male sterility lines currently used in broccoli breeding were derived from a line with dominant genic male sterility (DGMS), which was discovered in cabbage, and from a line Ogura-CMS, which was discovered in radish [8,9,10]. However, there are still some disadvantages regarding bud development and seed production in male sterility lines, especially in Ogura-CMS plants; these disadvantages include bud abortion and low seed yield [8,11,12]. On the other hand, hybrid cultivars from male sterility lines have enhanced seed purity and hybrid rates [11].

Transcriptome analyses based on RNA-sequencing (RNA-Seq) technology are important methods with which to understand the differentially expressed genes (DEGs) involved in various biological processes, and these analyses have been widely performed on *Brassica* plants [12,13,14]. For example, a small RNA-Seq has revealed DEGs in the early development of broccoli pollen [15], and transcriptome analyses have been used to examine four transcription factors in normal and abortive buds of an Ogura-CMS line and its maintainer line [12]. In addition, the roles of pigment mechanisms in post-harvest broccoli yellowing [16], gene expression patterns associated with sulforaphane metabolism in broccoli florets [17], glucosinolate metabolism in seeds and sprouts [18] and yield heterosis in curds of broccoli have also been elucidated by RNA-Seq technology [19]. So far, few studies on the mechanism of male sterility in broccoli have been reported. To the best of our knowledge, it would be the first report of comparative transcriptome analyses of isogenic Ogura-CMS, DGMS and inbred lines in broccoli florets.

To obtain a new male sterile line of broccoli for cultivar breeding, we used multiple broccoli inbred lines with cabbage DGMS material and developed several good DGMS broccoli lines with stable agronomic performance [8]. In the same way, Ogura-CMS broccoli lines derived from CMSR3629 (Ogura-CMS) introduced by the Asgrow Seed Co. (USA) have also been developed. Among these materials, the inbred line T54 (T54S) has selfed more than 10 generations. T54S was used to develop an isogenic Ogura-CMS line (T54C) and an isogenic DGMS line (T54M).

## 2. Results

### 2.1. Investigation of Agronomic Traits and the Nectary Morphology

**Figure 1:**
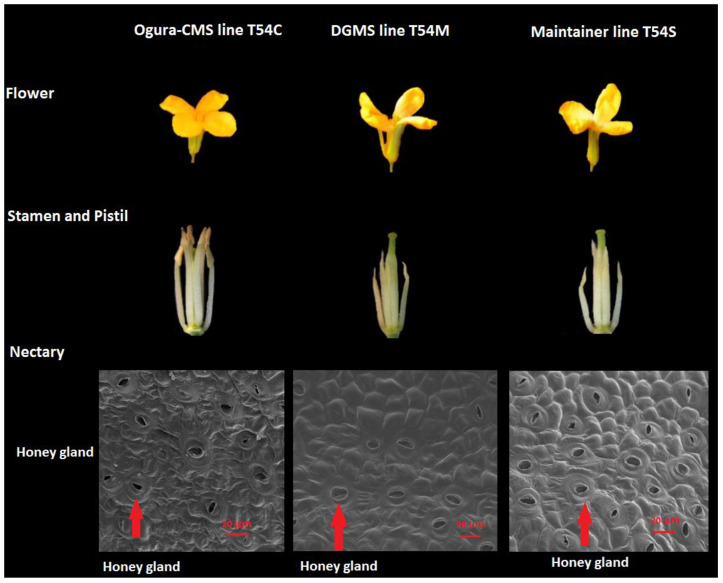
Agronomic traits of the flower, stamen and pistil, and the nectary morphology of the Ogura-CMS line T54C, the DGMS line T54M and their maintainer line T54S. The red arrows show the honey gland.

There were no significant differences in head characteristics among the Ogura-CMS line T54C, the DGMS line T54M and their maintainer fertile line T54S, but there were some other differences in fertility, maturity days, flowering time, production and seed characters, which are shown in and Figure 1 and Table 1. Agronomic traits of broccoli at harvest revealed that the Ogura-CMS broccoli showed stronger performance in plant height, leaf length, plant spread angle, single head weight, head width and stem diameter than the DGMS line or the maintainer fertile line. The DGMS line had longer production (days to maturation and flowering) than the Ogura-CMS and maintainer lines. In contrast, the maintainer fertile line presented a higher seed yield and seed germination rate than the DGMS and Ogura-CMS lines. The Ogura-CMS line showed the lowest seed yield and germination rate. From Figure 1, we could find more honey glands in the maintainer line than in the Ogura-CMS and DGMS lines, and most of them were open, while honey glands in the DGMS line was less significant than the other both lines.

### 2.2. Genomic Characteristicsand SNP Distribution

Six cDNA libraries from broccoli florets obtained at harvest times from the T54C, T54M and T54S lines were subjected to Illumina sequencing with two biological replicates for each sample. After the filtering of invalid reads, 159,424,568 clean reads and 47,827,370,400 clean bases (47.8 Gb) were obtained (Table S1), while the Q20 and Q30 percentages were 98.12–98.41% and 95.06–95.62%, respectively, with GC percentages of 45.08–46.80%. From Table S2, we found that eight regions of the genome contained Single-Nucleotide Polymorphism (SNP). The T54S line showed a higher percentage of SNP distribution in exonic regions (54.39%) than the T54C (52.35%) and T54M (52.79%) lines but a lower percentage in intergenic regions (15.85%) than the T54C (16.96%) and T54M (17.25%) lines. The remaining regions of SNP distribution showed no clear differences.

### 2.3. Gene Optimization and Correlation Test for Each Sample

A total of 5754 optimized regions involving 3872 unigenes were predicted using the gene structure optimization method, and approximately 13 mitochondrial unigenes (chrMT) were optimized. To study the reliability of the results and differences among the samples, correlation tests were performed for each sample comparison based on the log_2_ (FPKM) values of gene expression (Table 2 and Figure S1). All the coefficients of correlation for the pairs of samples ranged from 0.913 to 0.973, with the Pearson’s R^2^ test values ranging from 0.867 to 0.973 (R^2^ > 0.8). The coefficients of correlation among replicate T54C, T54M and T54S samples were 0.965 (R^2^ = 0.943), 0.960 (R^2^ = 0.925) and 0.966 (R^2^ = 0.919), respectively. The coefficients of correlation between T54C and T54M, T54C and T54S, and T54M and T54S ranged from 0.867 to 0.918, 0.867 to 0.905, and 0.894 to 0.973, respectively. Therefore, the DGMS and maintainer lines were more closely related than the Ogura-CMS and maintainer lines.

### 2.4. DEGs Analysis

**Figure 2:**
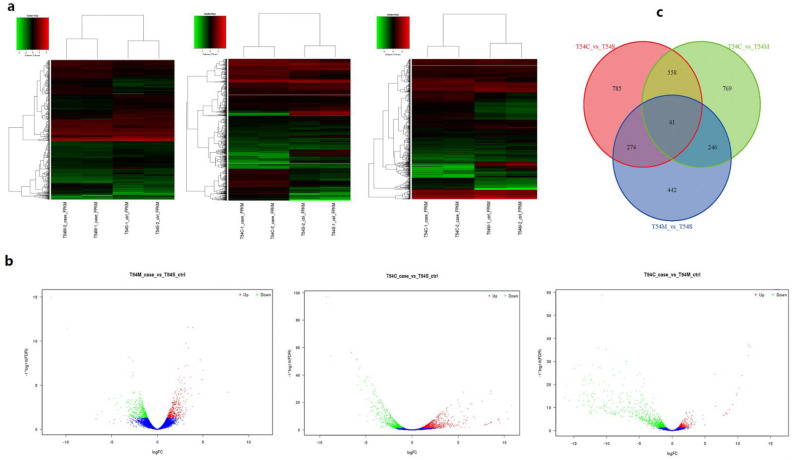
The DEGs identified in the Ogura-CMS line, DGMS and maintainer lines. (**a**) Clustering heat map of DEGs in T54C and T54S, T54C and T54M, and T54M and T54S, green and red colors indicated up- and downregulation of the genes, respectively. (**b**) Volcano plot of DEGs in comparisons of T54M vs. T54S, T54C vs. T54S, T54C vs. T54M, green and red colors indicated up- and downregulation of the genes, respectively. (**c**) The DEGs among broccoli lines of T54C vs. T54S, T54C vs. T54m, and T54M vs. T54S.

To explore the DEGs, the gene expression variations were analyzed in three comparisons T54C vs. T54M, T54C vs. T54S and T54M vs. T54S (Figure 2). In the Ogura-CMS line compared with the maintainer line, 585 and 1073 genes were up- and downregulated, respectively. In the DGMS line compared with the maintainer line, 469 and 534 genes were up- and downregulated, respectively. Moreover, we also compared the Ogura-CMS line to the DGMS line, 505 and 1109 up- and downregulated genes were identified, respectively. The DEG cluster analysis (Figure 2) revealed that the Ogura-CMS line showed similar up- and downregulated genes in comparison with the DGMS and maintainer lines, but the T54M vs. T54S comparison presented very different numbers and types.

The KEGG pathway results were comprehensively evaluated and filtered (*p* < 0.01), and there were eight pathways strongly related to plant development of the Ogura-CMS line, DGMS and maintainer lines. Specifically, 7 upregulated genes related with photosynthesis-antenna proteins, 41 upregulated genes related with plant hormone signal transduction, 39 downregulated genes related with phenylpropanoid biosynthesis, 4 up- and 5 downregulated genes related with adenosine triphosphate (ATP)-binding cassette (ABC) transporters, 5 upregulated genes related with fatty acid elongation, 25 downregulated genes related with fatty acid metabolism, 15 downregulated genes related with flavonoid biosynthesis and 8 upregulated genes related with polycyclic aromatic hydrocarbon degradation pathways were distributed among different samples, which provided potential genes in regulating morphological characteristics of the Ogura-CMS line, DGMS and inbred lines (Table S3).

### 2.5. Genes Related to Plant Hormones

**Figure 3:**
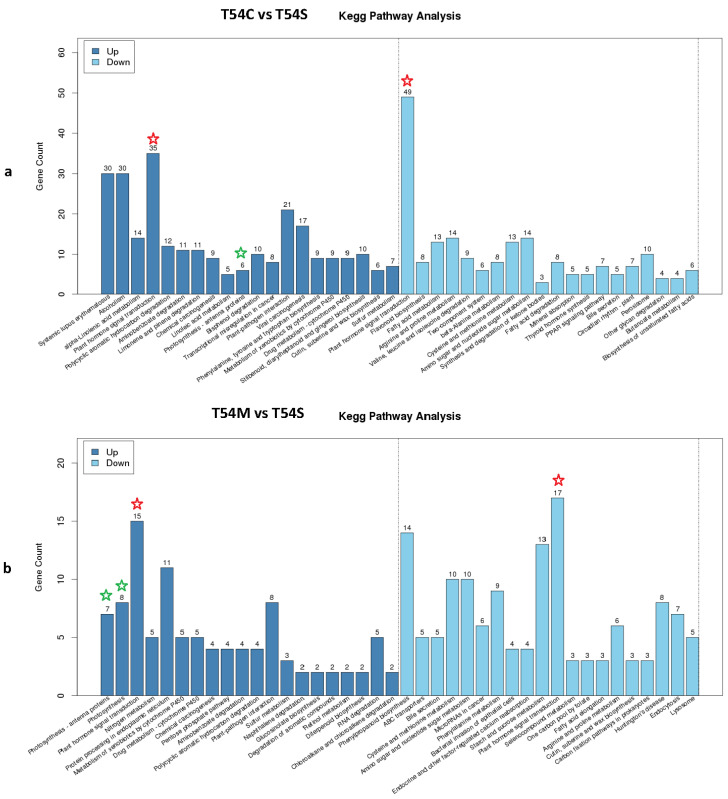
KEGG pathway analysis of the Ogura-CMS (**a**) and DGMS (**b**) lines vs. the maintainer line T54S. The red star stands for plant hormone signal transduction pathway and the green stars stands for photosynthesis-antenna proteins and photosynthesis pathways.

Plant hormones play important roles throughout the life span of a plant, such as auxin, gibberellin (GA), cytokinin (CK), abscisic acid (ABA), ethylene (ET), jasmonic acid (JA), salicylic acid (SA) and brassinosteroid (BR). The individual pathways of various plant hormone responses have been widely studied in the model organism Arabidopsis thaliana [20]. In this study, comparing to the maintainer line, in the plant hormone signal transduction pathway, 35 and 15 genes were upregulated in the Ogura-CMS and DGMS lines, respectively. Additionally, 49 and 17 genes were downregulated in the Ogura-CMS and DGMS lines, respectively (Figure 3). In the Ogura-CMS line T54C, these genes were associated with auxin, CK, ABA, ET, JA and SA metabolism. In the DGMS line T54M, they were obviously auxin genes (Aux/IAA and SAUR), CK-related gene (A-ARR), ABA-related gene (PP2C), ET-related gene (ERF1/2), BR-related gene (CYCD3) and JA-related gene (JAZ). Compared to the Ogura-CMS line T54C, the DGMS line T54M, there were common genes including auxin (Aux/IAA and SAUR), CK (A-ARR), ABA (PP2C), ET (ERF1/2), BR (CYCD3) and JA (JAZ). However, some others also exhibited in the DGMS line, with regard to auxin metabolism, two genes, TIR1 and GH3, were absent, and another similar gene, Aux/IAA, was present, which played a crucial role in repressing the expression levels of genes activated by auxin response factors (ARFs) in Arabidopsis [21].

### 2.6. Genes Related to Oxidative Phosphorylation and Photosynthesis

**Figure 4:**
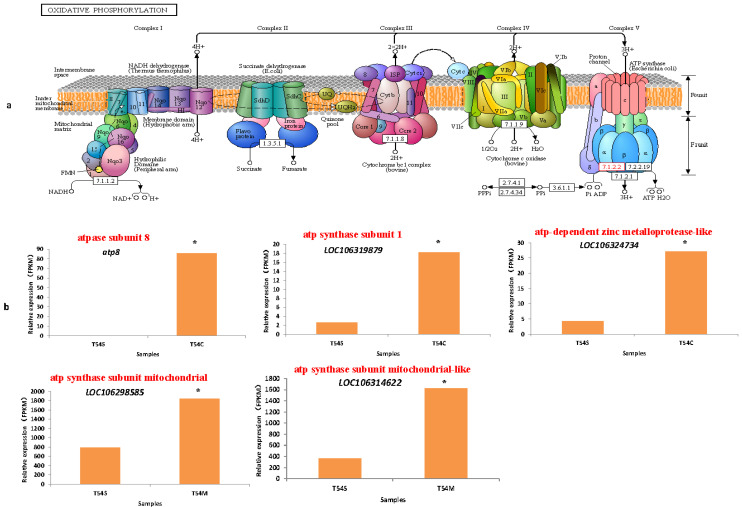
The oxidative phosphorylation pathway (**a**) and five upregulated DEGs with relative expression (FPKM) (**b**). The red frame in oxidative phosphorylation pathway represents upregulated genes (**a**) and red statements in (**b**) indicate the functional annotation. (*) indicated significant difference at *p* < 0.05 (**b**).

Mitochondrial Oxidative Phosphorylation (OXPHOS) provides Adenosine triphosphate (ATP) for driving cellular functions, which is of central importance for almost all eukaryotic cells. In plants, OXPHOS takes place in the condition of photosynthesis. Indeed, the metabolism of mitochondria and chloroplasts is tightly linked, and photosynthesis deeply affects OXPHOS [22]. In this study, 5 OXPHOS-upregulated genes of *atp8*, *LOC106319879*, *LOC106324734*, *LOC106314622* and *LOC106298585* were obviously found in the Ogura-CMS and DGMS lines, especially the atp8 gene just detected in the Ogura-CMS line with a higher relative expression level but nearly no expression in the maintainer line (Figure 4). From Figure 4, we could find the functions of these atp-related genes based on ko00190 (Oxidative phosphorylation) and ko00195 (Photosynthesis). At the same time, six genes related with photosynthesis-antenna proteins were upregulated in the Ogura-CMS line comparing to its maintainer line, while seven photosynthesis-antenna proteins and eight photosynthesis-related genes were upregulated in the DGMS line (Figure 4). According to our evaluation, three upregulated genes (Lhcb1, Lhcb3 and Lhcb5) associated with the photosynthesis-antenna protein pathway (ko00196) in the DGMS line were emphasized in this study.

### 2.7. Real-Time qPCR Validation of Gene Expression Patterns

**Figure 5:**
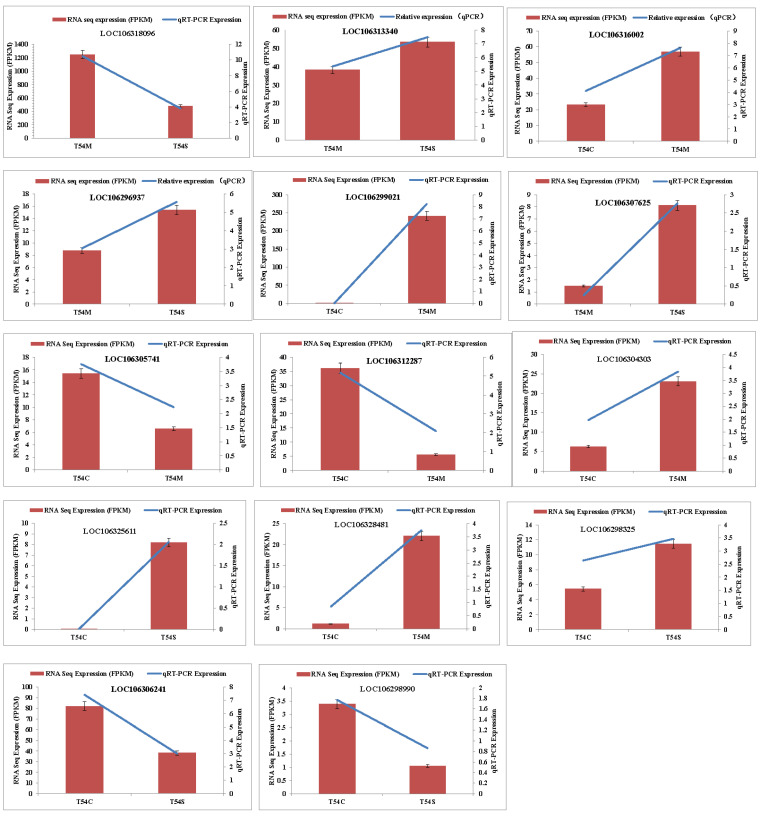
Verification of the selected DEGs carried out by qRT-PCR. Fourteen DEGs expression in T54S, T54C and T54M lines were selected for qRT-PCR validation. The relative expression level of each gene was shown as the FPKM between different two samples in the RNA-Seq data (red bar) and qRT-PCR results (blue line).

To better understand and explain the different agronomic traits of the broccoli lines T54S, T54M and T54C, the number of 14 DEGs were selected and their expression was validated by qRT-PCR (Figure 5). These genes were associated with photosynthesis-antenna proteins, plant hormone signal transduction, phenylpropanoid biosynthesis, ABC transporters, fatty acid elongation, fatty acid metabolism, flavonoid biosynthesis and polycyclic aromatic hydrocarbon degradation. One additional gene associated with each term was targeted, and the transcriptome data and qRT-PCR results showed similar patterns for these selected DEGs derived from RNA-sequencing analysis.

## 3. Discussion

### 3.1. Male Sterility in Brassica Crops

Male sterility lines, including Ogura-CMS and DGMS (especially Ogura-CMS), have been widely used in *Brassica* crops [8,12,23]. The Ogura-CMS line contributes significantly to hybrid seed production in *Brassica* crops. Therefore, some studies focus on Ogura-CMS, although the mechanism of hybrid seed production has not been elucidated. Previous transcriptome and proteome analyses of the Ogura-CMS line have provided basic information about the DEGs and differential abundant proteins (DAPs) between Ogura-CMS lines and their maintainer lines [12,13,14,24]. However, few studies focus on broccoli and the mechanism of male sterility in the Ogura-CMS, DGMS and maintainer lines. In addition, significant differences of some traits mentioned in this study, especially the maturity periods, flowering time and seed yield, do exist in practice which play an important role in broccoli breeding. Moreover, honey yield and the numbers of bees gathering honey in flowers are usually different in the male sterility and fertile crops [25]. However, there is still no comprehensive study on the mechanism of male sterility including the Ogura-CMS and DGMS lines and their maintainer line in broccoli. In this study, some important agronomic traits were noted and compared among the Ogura-CMS and DGMS lines and their maintainer line based on a two-year investigation. In contrast to previous studies, this study might elucidate the reasons of the stronger performance of plant height, largest leaf length, plant spread angle, single head weight, head width and stem diameter in the Ogura-CMS broccoli than in the DGMS line, and why the DGMS broccoli had longer maturation and flowering periods than the Ogura-CMS and inbred lines. However, the Ogura-CMS broccoli was poorer in seed yield and germination rate than the maintainer line.

### 3.2. Plant Hormone Signal Transduction and ABC Transporters Affected the Growth Performance and Reproductive Traits

As is known to all, plant hormones play important roles in plant growth and development. In this study, the plant hormone signal transduction and ABC transporters pathways were both significantly enriched for DEGs in the Ogura-CMS line compared to the DGMS and maintainer lines, respectively. To better understand the morphological characterization difference in three broccoli lines, we explored the possible molecular mechanism associated with DEGs of plant hormones.

**Figure 6:**
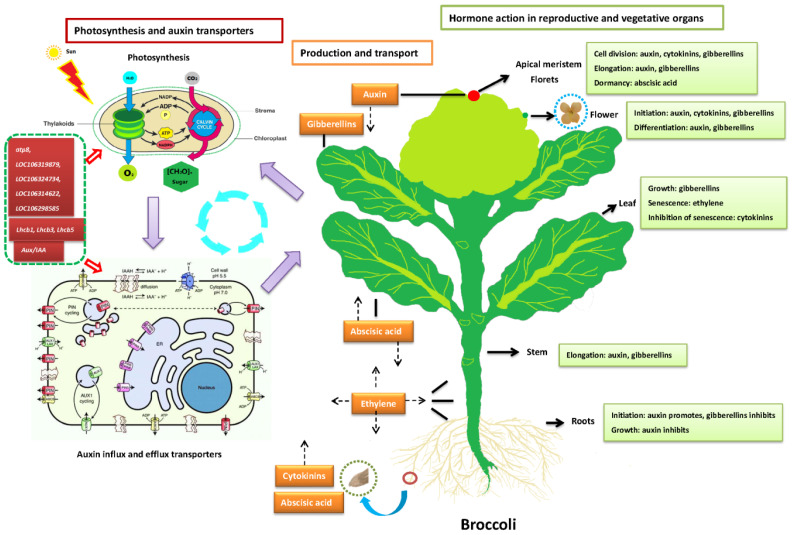
The role of photosynthesis, auxin transporters and plant hormones in reproductive and vegetative organs of broccoli. The genes in stuffed red frames surrounded by green dotted line were from pathways of OXPHOS (5), photosynthesis-antenna protein (3) and plant hormones (1). The orange box indicates plant hormones and the dummy arrow shows their direction of transport, while the black line indicates their synthetic organs or tissues. The stuffed olive frames indicate the functions of plant hormones in different organs and tissues. The red round stands for apical point of broccoli and green round in florets stands for one bud.

Auxin, the polar distribution of the plant hormone, is unique and the primary cause for the establishment of local auxin maxima and minima that trigger a plethora of physiological and developmental programs [26]. The generation, control and flexibility of these gradients require the coordinated action of more than three major auxin transporter families including the PIN-FORMED (PIN), the AUXIN1-RESISTANT1 (AUX1)/LIKE AUX1 (AUX1/LAX) and the ABC transporters of the B family (ABCBs) [27,28,29] (Figure 6). Additionally, the subgroup of auxin-transporting ABCBs functions as primary active (ATP-dependent) auxin pumps could transport against steep auxin gradients [30,31]. Thus, ATP is quite necessary for the auxin influx and efflux transporters, and photosynthesis is the initial and powerful basis for the growth and development of plants, especially in plant hormone signal transduction and ABC transporters which could strongly affect all kinds of reproductive and vegetative organs in broccoli and the other eukaryotes [32] (Figure 6). In our study, a number of plant hormone genes of auxin (Aux/IAA and SAUR), CK (A-ARR), ABA (PP2C), ET (ERF1/2), BR (CYCD3) and JA (JAZ) had been detected and highly upregulated in the Ogura-CMS and DGMS lines compared to the maintainer lines, repeatedly. Meanwhile, five OXPHOS-upregulated genes of *atp8*, *LOC106319879*, *LOC106324734*, *LOC106314622* and *LOC106298585* were also obviously found in the Ogura-CMS and DGMS lines (Figure 6). So, we declared that those functional genes associated with plant hormones and OXPHOS might provide a good explanation for the differences in morphological and transcriptomic characterization in the Ogura-CMS, DGMS and maintainer broccoli lines, and as well as some other Brassica crops.

In addition, with regard to auxin metabolism, the *TIR1*, *GH3* and *SAUR* families were all obviously upregulated, which could promote cell enlargement during plant growth promotion. The *SCF* (*TIR1*) *E3* ubiquitin ligase complex is involved in the auxin-mediated signaling pathway that regulates root and hypocotyl growth, lateral root formation, cell elongation and gravitropism. The auxin-responsive *GH3* family is essential for plant growth and development in rice, and several genes of this family have been proved to have indoleacetic acid (IAA) amido synthetase biochemical activity [33,34]. The *SAUR* gene family was initially defined as a set of auxin-inducible genes regulating development, especially in hypocotyls, and many *SAUR* genes act by promoting cell expansion [35,36]. Additionally, the *NPR1* gene associated with SA metabolism was highly upregulated in the Ogura-CMS line rather than the DGMS and maintainer lines, so we inferred that the *NPR1* gene might be a domain regulator in the SA-mediated systemic acquired resistance (*SAR*) pathway and play an essential role in plant immunity [37]. In *Arabidopsis*, *NPR1* can interact with transcription factors to induce the expression of pathogenesis-related (*PR*) genes and strongly promote defense responses [38], which was consistent with its agronomic characters in this study.

### 3.3. The DGMS Line Responds to Plant Hormone Signal Transduction

Notably, *Arabidopsis* plants overexpressing *CYCD3-1* show extensive leaf curling, disorganized meristems, increased leaf numbers, late flowering and delayed senescence [39]. In our study, the *CYCD3* gene associated with BR metabolism was upregulated in the DGMS broccoli line, which might be an important find for explaining longer maturation and flowering periods than the Ogura-CMS and maintainer fertile lines. Additionally, the J-related gene (*JAZ*) was present in the DGMS line, while two JA-related genes (*JAZ* and *MYC2*) were present in the Ogura-CMS line, which might be act as reliable evidence to explaining the better seed yields in the DGMS line. No SA gene upregulation was found in the DGMS line. Therefore, we found that the BR gene *CYCD3* might function in late flowering and delayed senescence of broccoli during the growth and developmental stages consistent with the investigation data obtained over two ecotypes.

## 4. Materials and Methods

### 4.1. Plant Materials and Investigation of Agronomic Traits

The broccoli (*Brassica oleracea* var. *italica*) maintainer fertile T54S (inbred line F_10_) has selfed more than 10 generations, and the Ogura-CMS T54C derived from CMSR3629 (Ogura-CMS) introduced by the Asgrow Seed Co. (USA) have also been developed [40,41]. Additionally, the DGMS line T54M was initially derived from the cabbage line 79-399-3 [8,42]. All the materials were bred at the Institute of Vegetables and Flowers, Chinese Academy of Agricultural Sciences (IVF-CAAS). In the autumn of 2019 and 2020, three replicates with the plant spacing 50 × 45 cm^2^, were successively grown in the same experimental greenhouse on August 12 at IVF-CAAS (Beijing, China) (40°15′ N, 116°83′ E). When plant harvest began on October 10, three types of bud samples were typically collected from the middle and around the head (diameter 14–15 cm) of T54S, T54M and T54C and labeled with two biological replicates. The isolated buds were immediately frozen in liquid nitrogen and stored at −80 °C for use.

### 4.2. Scanning Electron Microscopy

The completely open flowers derived from T54S, T54M and T54C, were dissected and dehydrated through a series of increasing ethanol solutions. Additionally, the materials were then critically point-dried with solvent-substituted liquid carbon dioxide and coated with a thin layer of gold palladium. Photomicrographs were obtained using a JEOL 5800 LV at 20 kV (JEOL USA, Peabody, MA, USA). At maturity, the nectaries of broccoli at bolting stage were cut off and pre-fixed in 1% glutaraldehyde and 4% formaldehyde in phosphate buffer (pH 7.2) for 4 h. The nectaries were then post-fixed in 1.5% O_s_O_4_ at 4 °C in the same buffer for 4 h, and then, the samples were dehydrated in ascending graded series of acetone and embedded in Spurr’s resin. Ultrathin sections were made using a Reichert ultramicrotome and stained with uranyl acetate and lead citrate [43,44]. The equipment of JEOL-JEM 1200 Ex II transmission electron microscopy (TEM) was used for identifying all the samples at 85 kV.

In addition, the agronomic traits of the broccoli lines T54S, T54M and 54C were investigated during the harvest, flowering, seed ripening and seed germination stages. All the data were analyzed with SPSS 19.0 software (IBM Co., Ltd., New York, NY, USA), and one-way ANOVA was carried out on the agronomic trait data at *p* < 0.05.

### 4.3. RNA Extraction and Illumina Sequencing

Total RNA was extracted using an RNA Pure Plant Kit (Tiangen Co., Ltd., Beijing, China) following the manufacturer’s instructions. The integrity of the RNA was assessed with an RNA Nano 6000 Assay Kit on an Agilent Bioanalyzer 2100 platform (Agilent Technologies Inc., Santa Clara, CA, USA). High-quality RNA from each sample was used for cDNA library construction and RNA-Seq on an Illumina HiSeq^TM^ 2500 platform (Berry Genomics Co., Beijing, China). To obtain clean high-quality reads, adapter sequences, low-quality reads (in which >50% bases had Q-values ≤3) and unknown sequences (“N” reads > 10% unknown nucleotides) were removed from the raw reads. The Q20, Q30 and GC contents were then calculated based on the clean reads.

### 4.4. Quality Control and Transcriptome Analysis

The clean reads from each sample were mapped to the reference genome (ftp://brassicadb.org/Brassica_oleracea/) with TopHat2 (Version 2.0.3.12) [45]. The gene expression levels were normalized using the fragments per kilobase of transcript per million mapped reads (FPKM) method. The edgeR package (Version 2.15.2) was used to identify differentially expressed genes (DEGs) between two samples. Each gene was quantified by calculating the FPKM value with RSEM (V1.2.15). Thresholds of an FDR < 0.05 and a log2 |fold change| >1 were set to select the DEGs. The DEGs were then analyzed for enrichment of Gene Ontology functions and Kyoto Encyclopedia of Genes and Genomes (KEGG) pathways [17]. GO terms or pathways with *p* values <0.05 were defined as significantly enriched for the DEGs. Correlations among the expression levels of DEGs were analyzed with Pearson correlation tests [46].

### 4.5. Quantitative qRT-PCR Analysis

Quantitative real-time (qRT-PCR) analyses were performed to validate the results from the DEGs with the three biological replicates. These are the results of the study: fourteen DEGs were chosen and tested, and six of them were specifically upregulated, and eight of them were specifically downregulated in T54M vs. T54S samples, T54C vs. T54M samples and T54C vs. T54S samples. Total RNA was extracted from the head buds of each sample at harvest time using an RNA Pure Plant Kit (Tiangen Co., Ltd., Beijing, China). First-strand cDNA was synthesized using a PrimeScript^TM^ 1st Strand cDNA Synthesis Kit (Takara, Dalian, China). qRT-PCR was carried out according to the SYBR PrimeScript RT-PCR Kit manufacturer specifications (Takara, Dalian, China) on an ABI Prism^®^ 7900HT Real-Time PCR System (Applied Biosystems, Foster City, CA, USA). Three biological replicates (with three technical replicates for each biological replicate) were analyzed for each gene, and the relative expression level was estimated by the 2^-ΔΔCt^ method [47,48]. The *β-actin* gene was used as an internal control, and the specific primers were designed using Primer Premier 6 (Premier Co., Palo Alto, PA, Canada) (Table 3) [47].

### 4.6. Statistical Analysis

All data were analyzed by analysis of variance (ANOVA) using SPSS Statistics version 19.0 (SPSS, Inc., Chicago, IL, USA) and were expressed as the mean ± standard errors. One-way ANOVA and Tukey’s multiple-range test were used to evaluate the significant differences (*p* < 0.05). The statistical analyses and charts were produced using the IBM SPSS software and GraphPad Prism 8.2.0 (GraphPad Software Inc., San Diego, CA, USA), respectively.

## 5. Conclusions

In this study, we firstly significant differences of reproductive traits did exist in three isogenic lines. A total of 5 OXPHOS genes might play role in reproductive traits of the Ogura-CMS broccoli. Meanwhile, 3 photosynthesis genes were found to be highly relative with flowering time in the DGMS broccoli. Our research would provide a comprehensive foundation for understanding the differences of electrophysiological, morphological and transcriptomic profiles in the Ogura-CMS, DGMS and maintainer broccoli, and as well as beneficial to exploring the mechanism of male sterility in *Brassica* crops.

## Figures and Tables

**Table 1:** Investigation on agronomic traits of broccoli lines T54S, T54M and T54C in the autumn of 2019 and 2020.

Trait Name	2019			2020		
	T54S	T54M	T54C	T54S	T54M	T54C
Plant height (cm) *	72.48 ± 4.25 b	70.33 ± 4.29 c	73.27 ± 3.14 a	68.67 ± 3.65 a	65.39 ± 3.74 c	68.18 ± 2.85 b
Length of the largest leaf (cm) *	60.37 ± 2.49 b	58.56 ± 3.87 c	61.48 ± 3.04 a	58.88 ± 3.07 b	56.49 ± 2.28 c	59.71 ± 3.63 a
Width of the largest leaf (cm)	25.28 ± 2.79 a	24.17 ± 1.89 b	25.08 ± 2.12 a	24.22 ± 1.75 b	24.01 ± 2.01 b	25.53 ± 2.28 a
Plant spread angle (cm) *	91.04 ± 5.85 b	86.34 ± 6.15 c	92.86 ± 6.19 a	88.18 ± 5.35 b	86.27 ± 6.05 c	90.84 ± 7.56 a
Single head weight (kg) *	0.24 ± 0.04 b	0.23 ± 0.07 c	0.26 ± 0.04 a	0.22 ± 0.05 b	0.21 ± 0.08 c	0.24 ± 0.05 a
Head height (cm)	15.24 ± 1.16 a	15.26 ± 2.06 a	15.36 ± 2.18 a	15.11 ± 1.89 a	14.84 ± 1.78 a	15.26 ± 2.24 a
Head width (cm) *	15.37 ± 1.55 b	15.22 ± 2.08 b	16.04 ± 2.57 a	15.08 ± 1.87 a	14.39 ± 2.06 b	15.53 ± 3.15 a
Stem diameter (cm) *	5.12 ± 0.18 a	4.98 ± 0.27 b	5.16 ± 0.42 a	4.87 ± 0.38 a	4.35 ± 0.18 b	4.96 ± 0.49 a
Stem hollow/solid	Solid	Solid	Solid	Solid	Solid	Solid
Leaf number	10 ± 2 a	9 ± 2 a	10 ± 2 a	9 ± 1 a	10 ± 3 a	9 ± 2 a
Leaf color	Green	Green	Green	Green	Green	Green
Leaf waxiness	High	High	High	High	High	High
Flower color	Yellow	Yellow	Yellow	Yellow	Yellow	Yellow
Days to maturity (day)*	105 ± 3 b	109 ± 4 a	106 ± 4 b	126 ± 3 b	134 ± 4 a	128 ± 4 b
Days to flowering (day) *	126 ± 3 b	129 ± 3 a	126 ± 4 b	143 ± 2 b	147 ± 3 a	142 ± 2 b
Seeds yield per plant (g) *	4.13 ± 0.24 a	3.52 ± 0.58 b	2.41 ± 0.27 c	2.86 ± 0.29 a	2.21 ± 0.56 b	1.74 ± 0.36 c
Seeds germination rate (%) *	86.58 ± 3.77 a	83.04 ± 4.18 b	71.76 ± 2.47 c	85.03 ± 2.61 a	79.59 ± 2.92 b	71.46 ± 2.88 c

Note: T54C, T54M and T54S represented samples from the Ogura-CMS, DGMS and maintainer lines, respectively. All the data were shown as the mean ± standard deviation (n = 3). Days to maturity represented the number of days from sowing to head maturity, and days to flowering represented the number of days from sowing to flowering. The asterisk (*) and minuscule show that a significant difference was identified among the T54S, T54M and T54C groups in the same season based on one-way ANOVA at *p* < 0.05.

**Table 2:** Correlation analysis of gene expression levels between replicate samples of broccoli.

Serial Number	Sample Name	Linear Equation	Pearson Test (R^2^)
1	T54C-1 vs. T54C-2_FPKM	Y = 0.965*X + 0.025	0.943
2	T54C-1 vs. T54M-1_FPKM	Y = 0.939*X − 0.053	0.918
3	T54C-1 vs. T54M-2_FPKM	Y = 0.915*X + 0.046	0.875
4	T54C-1 vs. T54S-1_FPKM	Y = 0.913*X + 0.177	0.867
5	T54C-1 vs. T54S-2_FPKM	Y = 0.937*X + 0.150	0.890
6	T54C-2 vs. T54M-1_FPKM	Y = 0.945*X − 0.073	0.918
7	T54C-2 vs. T54M-2_FPKM	Y = 0.917*X + 0.028	0.867
8	T54C-2 vs. T54S-1_FPKM	Y = 0.918*X + 0.158	0.867
9	T54C-2 vs. T54S-2_FPKM	Y = 0.946*X + 0.130	0.905
10	T54M-1 vs. T54M-2_FPKM	Y = 0.960*X + 0.099	0.925
11	T54M-1 vs. T54S-1_FPKM	Y = 0.947*X + 0.231	0.896
12	T54M-1 vs. T54S-2_FPKM	Y = 0.973*X + 0.205	0.973
13	T54M-2 vs. T54S-1_FPKM	Y = 0.947*X + 0.141	0.894
14	T54M-2 vs. T54S-2_FPKM	Y = 0.967*X + 0.013	0.917
15	T54S-1 vs. T54S-2_FPKM	Y = 0.966*X − 0.013	0.919

Note: T54C, T54M and T54S represented the samples of the Ogura-CMS, DGMS and maintainer lines, respectively. Y and X represented the values of log_2_ (FPKM) between two samples, and * indicates that correlation was highly significant between two samples at *p* < 0.05.

**Table 3:** Genes targeted by the specific primers for qRT-PCR analysis.

Gene	Function Description	Chromosome	Forward Primer 5′-3′	Reverse Primer 5′-3′	Ta (°C)
*LOC106318096*	Chlorophyll a-b binding protein 13, chloroplastic	C9	TCTCCACCAAACCAGCAAAG	TCAAGACCGTTGATGCGGAA	59
*LOC106313340*	Auxin-responsive protein IAA4	C9	CTTGGGTTTTCGGGGGAAGA	CCGTTTAAGCCTCTCACTGGT	60
*LOC106316002*	Ethylene receptor 2 (*BO-ETR2*)	C1	TGGGTTTAAGCATTTTCATGGGA	GAAAGGGTGGGGACCGTAAG	60
*LOC106296937*	Cinnamoyl-CoA reductase 2-like	C6	AACGGAGCCAAGTTCGTGAT	CGAAAAGAAGAGGGCATTCAGC	60
*LOC106299021*	Cytochrome P450 98A8-like	C6	TGGTGGGCATCCAACATACC	TTGTCGCTAACGAGCCACTT	60
*LOC106307625*	ABC transporter B family member 2	C1	GGAGGCGTCCAGTGATCC	TCCAACGAGTACTTGGCGAC	60
*LOC106305741*	ABC transporter B family member 19	C7	AGATCATCCGGGGAGGTGAA	TCATCAAGCATCAAATCATAAGCGT	60
*LOC106312287*	3-ketoacyl-CoA synthase 1 (KCS)	C8	GCCGTCTCTATCGGCAATGA	CACTCCACATATCCGGTCCA	60
*LOC106304303*	Palmitoyl-protein thioesterase 1	C7	TTCGGGTTTTACCCGGATGG	TACGCAAAGGCTCCTTGGTT	60
*LOC106325611*	3-oxoacyl-[acyl-carrier-protein] synthase II, chloroplastic-like	C2	TGGCTGCCTCTTCCTGTTAC	CCGGAGTTAGTTGCTCGGTT	59
*LOC106328481*	Chalcone synthase 3-like	C3	CCAAGCTCCTTGGTCTTCGT	ACGATGCTGGGAAGTATGTGT	59
*LOC106298325*	Chalcone--flavonone isomerase-like	C6	TTGGCTACCTACCTCCTCCC	TCAAACGAACCCGTGACGAT	60
*LOC106306241*	Cytochrome P450 81D11	C7	ACGAATCTGCGAAGGTGGAG	TGCCTGCATCTGCGGAATAA	61
*LOC106298990*	Cytochrome P450 71B5	C6	CCGACCTAAGACGGTAGGGA	CCGGAGATCCGGTCAATGAG	60
*β-actin*	Beta-actin		ATCTGGCATCACACTTTCTAC	ATCTCTTTGCTCATACGGTCT	55

## Data Availability

The data presented in this study are available on request from the corresponding author.

## Supplementary Materials

The following supporting information can be downloaded at: https://www.mdpi.com/article/10.3390/plants11040561/s1, Table S1: Summary of the transcriptome sequencing data, Table S2: The KEGG pathways enriched for the DEGs, Table S3: SNP distribution in genomic regions of the Ogura-CMS, DGMS and maintainer broccoli lines, Figure S1: Correlation analysis of gene expression levels between replicate samples of broccoli.
