# Supplementary material for: Electrophysiological, Morphologic, and Transcriptomic Profiling of the Ogura-CMS, DGMS and Maintainer Broccoli Lines
Source: Plants (Basel). 2022 Feb 21;11(4):561. doi: 10.3390/plants11040561 (PMC8880064; doi:10.3390/plants11040561)

Figure S1: Correlation analysis of gene expression levels between replicate samples of broccoli.

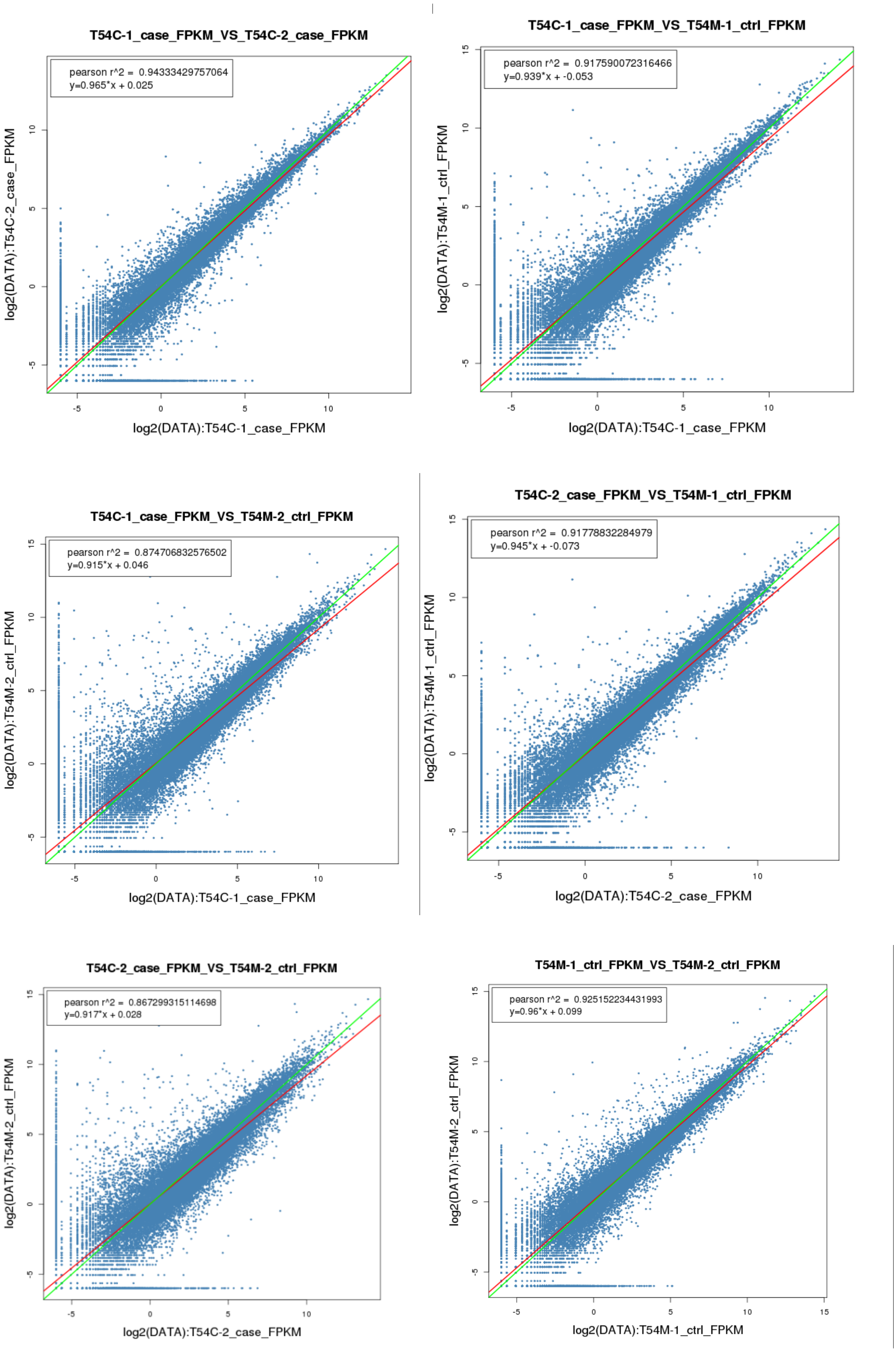

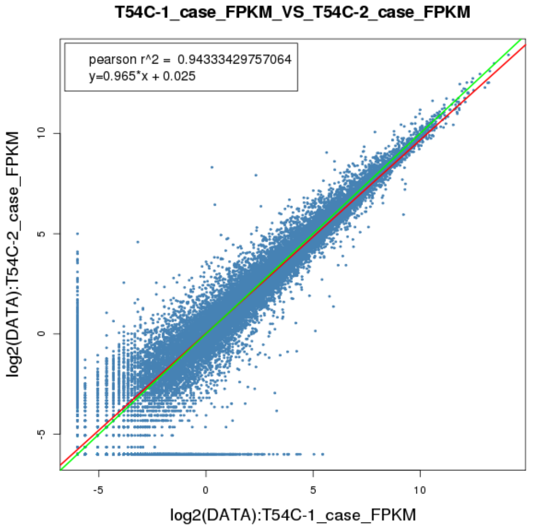
T54C-1_case_FPKM_VS_T54C-2_case_FPKM
pearson r^2 = 0.94333429757064
y=0.965*x + 0.025
log2(DATA):T54C-2_case_FPKM
log2(DATA):T54C-1_case_FPKM

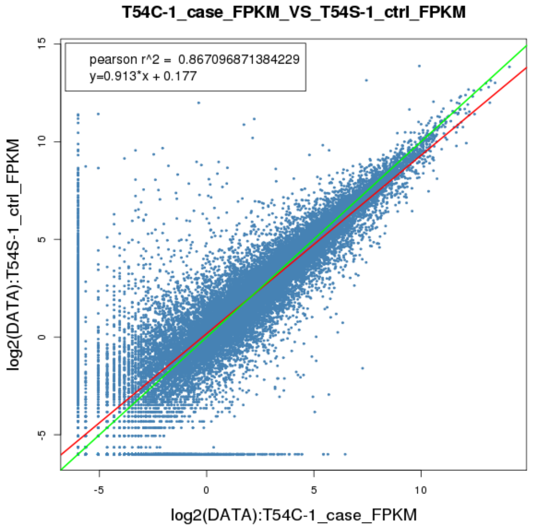
T54C-1_case_FPKM_VS_T54S-1_ctrl_FPKM
pearson r^2 = 0.867096871384229
y=0.913*x + 0.177
log2(DATA):T54S-1_ctrl_FPKM
log2(DATA):T54C-1_case_FPKM

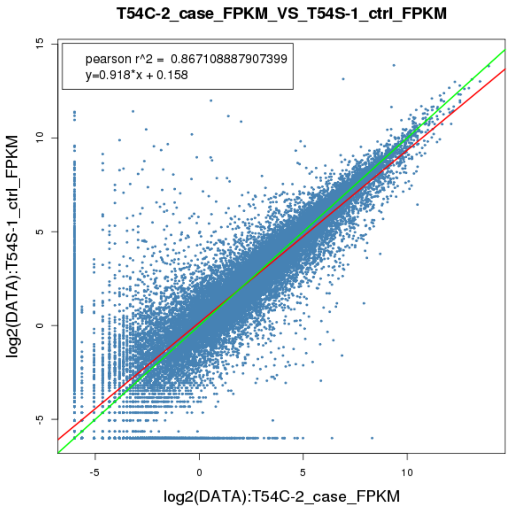
T54C-2_case_FPKM_VS_T54S-1_ctrl_FPKM
pearson r^2 = 0.867108887907399
y=0.918*x + 0.158
log2(DATA):T54S-1_ctrl_FPKM
log2(DATA):T54C-2_case_FPKM

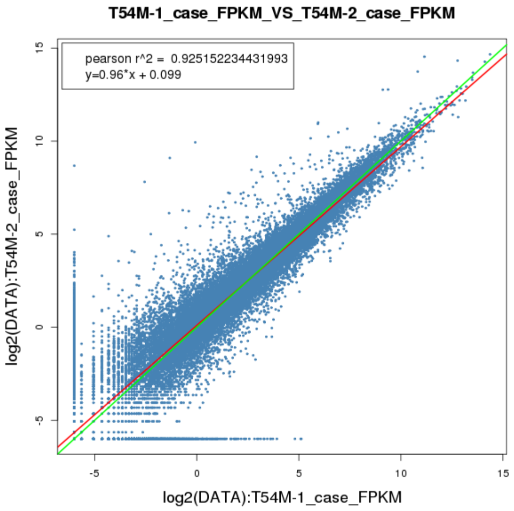
T54M-1_case_FPKM_VS_T54M-2_case_FPKM
pearson r^2 = 0.925152234431993
y=0.96*x + 0.099
log2(DATA):T54M-2_case_FPKM
log2(DATA):T54M-1_case_FPKM

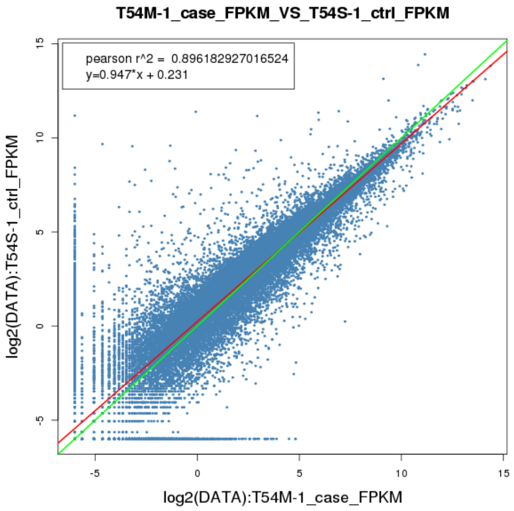
T54M-1_case_FPKM_VS_T54S-1_ctrl_FPKM
pearson r^2 = 0.896182927016524
y=0.947*x + 0.231
log2(DATA):T54S-1_ctrl_FPKM
log2(DATA):T54M-1_case_FPKM

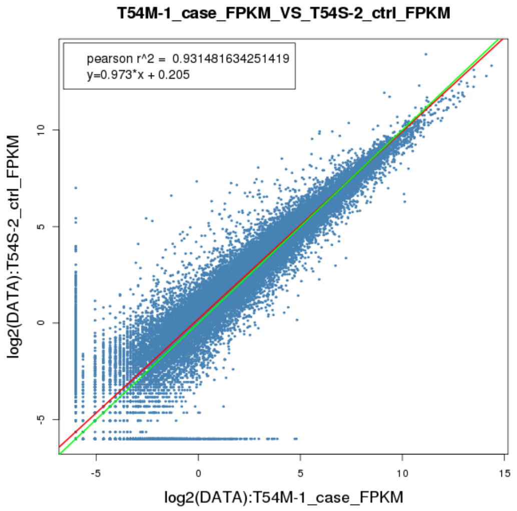
T54M-1_case_FPKM_VS_T54S-2_ctrl_FPKM
pearson r^2 = 0.931481634251419
y=0.973*x + 0.205
log2(DATA):T54S-2_ctrl_FPKM
log2(DATA):T54M-1_case_FPKM

T54M-2_case_FPKM_VS_T54S-1_ctrl_FPKM

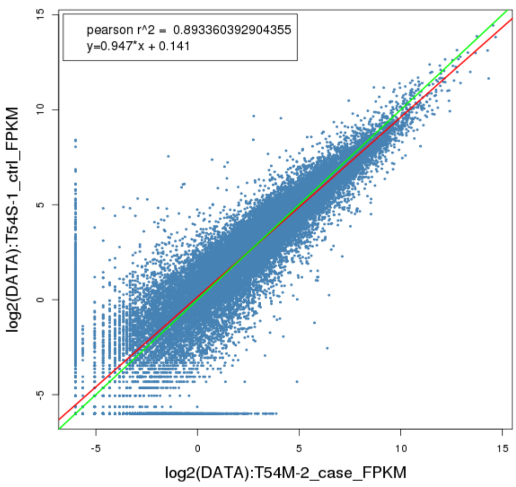

T54M-2_case_FPKM_VS_T54S-2_ctrl_FPKM

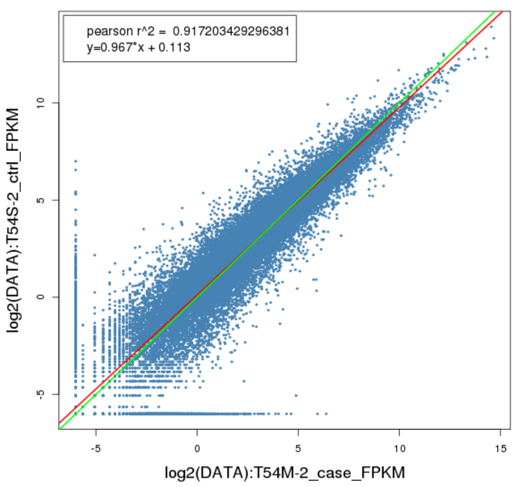

T54S-1_ctrl_FPKM_VS_T54S-2_ctrl_FPKM

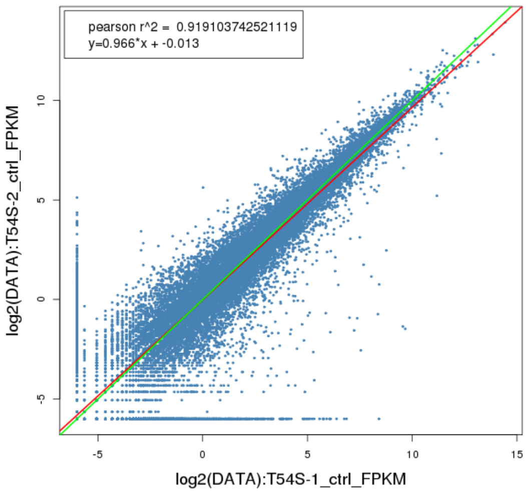

Supplement: Supplementary file 1 [file plants-11-00561-s001.zip › Figure S1.pdf]
