# Supplementary material for: Electrophysiological, Morphologic, and Transcriptomic Profiling of the Ogura-CMS, DGMS and Maintainer Broccoli Lines
Source: Plants (Basel). 2022 Feb 21;11(4):561. doi: 10.3390/plants11040561 (PMC8880064; doi:10.3390/plants11040561)
Supplement: Supplementary file 1 [file plants-11-00561-s001.zip › Tables S1-3.pdf]

Table S1. Summary of the transcriptome sequencing data.

Sample Name	Clean Reads	Clean Bases (Gb)	Clean N (%)	Clean GC (%)	Clean Q20 (%)	Clean Q30 (%)	Mapped Reads (Mapped/Clean)
**T54C-1**	30962081	9.29	0.00	46.74	98.34	95.47	72.70%
**T54C-2**	27469179	8.24	0.00	46.80	98.2	95.11	72.30%
**T54M-1**	28243036	8.47	0.00	46.56	98.34	95.48	73.00%
**T54M-2**	24472981	7.34	0.00	45.08	98.12	95.06	71.60%
**T54S-1**	25153030	7.55	0.00	46.27	98.33	95.43	72.60%
**T54S-2**	23124261	6.94	0.00	46.63	98.41	95.62	72.90%

Note: T54C, T54M and T54S represented the samples of the Ogura-CMS, DGMS and maintainer lines, respectively.

Table S2. The KEGG pathways enriched for the DEGs.

Term	Sample	ID	Input No	Background No	*p*-Value	Corrected *p*-Value	Up/Down
**Photosynthesis -antenna proteins**	T54M vs T54S	ko00196	7	35	0.00000	0.00004	up
**Plant hormone signal transduction**	T54M vs T54S	ko04075	15	548	0.00185	0.05459	up
	T54C vs T54S	ko04075	26	548	0.00000	0.00009	up
**Phenylpropanoid biosynthesis**	T54M vs T54S	ko00940	14	272	0.00025	0.02413	down
	T54C vs T54S	ko00940	25	272	0.00000	0.00019	down
**ABC transporters**	T54M vs T54S	ko02010	5	33	0.00036	0.02413	down
	T54C vs 54M	ko02010	4	33	0.00145	0.06517	up
**Fatty acid elongation**	T54C vs 54M	ko00062	5	41	0.00036	0.04808	up
**Fatty acid metabolism**	T54C vs 54M	ko01212	12	127	0.00036	0.03038	down
	T54C vs T54S	ko01212	13	127	0.00015	0.00754	down
**Flavonoid biosynthesis**	T54C vs 54M	ko00941	7	45	0.00045	0.03038	down
	T54C vs T54S	ko00941	8	45	0.00010	0.00680	down

**Polycyclic aromatic hydrocarbon degradation**	T54C vs T54S	ko00624	8	110	0.00072	0.01574	up

Table S3. SNP distribution in genomic regions of the Ogura-CMS, DGMS and maintainer broccoli lines.

Sample Name	Exonic	Splicing Site	5′ UTR	3′ UTR	Intronic	Upstream	Downstream	Intergenic
**T54C-1**	52.66%	0.40%	6.63%	9.82%	7.60%	2.32%	3.71%	16.86%
**T54C-2**	52.92%	0.40%	6.60%	9.61%	7.72%	2.25%	3.45%	17.05%
**T54M-1**	53.29%	0.40%	6.32%	10.10%	7.11%	2.24%	3.59%	16.95%
**T54M-2**	51.41%	0.35%	5.52%	10.89%	7.52%	2.44%	4.33%	17.55%
**T54S-1**	53.57%	0.40%	6.52%	10.18%	7.66%	2.12%	3.42%	16.13%
**T54S-2**	55.21%	0.41%	6.66%	9.98%	7.01%	2.06%	3.10%	15.56%

Note: T54C, T54M and T54S represent the samples of the Ogura-CMS, DGMS and maintainer lines, respectively.
